# Clinical and sociodemographic characterization of a sibling-matched cohort of children with autism Spectrum disorder in Uruguay

**DOI:** 10.3389/frcha.2026.1835921

**Published:** 2026-05-29

**Authors:** Lucia Lamberti, Camila Rusiñol, Andreina Guisande, Florencia Peñalba, Maite Irastorza, Florencia Konik, Andrés Parada, Claudio Iglesias, Paula Mendive, Nadia Riera, Gabriela Garrido

**Affiliations:** 1 Academic Unit of Pediatric Psychiatry, School of Medicine, University of the Republic, Montevideo, Uruguay.; 2 Department of Pediatric Gastroenterology, Hepatology and Nutrition, Pereira Rossell Hospital Center, Montevideo, Uruguay; 3 Microbial Genomics Laboratory, Institut Pasteur de Montevideo, Montevideo, Uruguay.; 4 Unidad Académica Área de Investigación, Escuela de Nutrición, Universidad de la República, Montevideo, Uruguay; 5 Center for Innovation in Epidemiological Surveillance, Institut Pasteur Montevideo, Montevideo, Uruguay.

**Keywords:** autism spectrum disorder, developmental milestones, dietary intake, gastrointestinal symptoms, sociodemographics

## Abstract

This study characterized clinical, sociodemographic, psychiatric, and gastrointestinal variables in 55 families, comparing children with Autism Spectrum Disorder (ASD) to neurotypical siblings. Primary objectives were to delineate sociodemographic profiles and systemic barriers, establish a clinical-nutritional baseline for Uruguayan children, and generate metadata for future microbiome research. We conducted a cross-sectional, case-control study including children aged 4–10 years and sibling controls. Professionals collected data via clinical interviews. Findings indicate a higher male prevalence in the ASD group. Mothers predominantly participated in caregiving and interviews. In the ASD group, 60% of pregnancies had complications, including 12 twin pregnancies. No significant differences were found regarding parental age, delivery method, prematurity, or birth anthropometry. Head circumference at birth did not associate with regression or severity. All children with ASD showed sensory particularities; 94.3% reported gastrointestinal symptoms. We observed a 20-month diagnostic gap and 65% regression rates between 12 and 36 months. These data highlight the need for improved early detection and provide essential local evidence for the Uruguayan ASD population. This interdisciplinary approach bridges clinical practice and research, advancing contextualized care in Uruguay.

## 1 Introduction

Neurodevelopment is a complex, lifelong process involving the maturation of the Central Nervous System (CNS) through the interaction of genetics, environment, and experience (1). Disruptions in this maturation can lead to neurodevelopmental disorders, manifesting as atypical cognitive and behavioral patterns (2). Among these, Autism Spectrum Disorder (ASD) is characterized by persistent challenges in social communication and the presence of restrictive, repetitive behaviors or sensory processing sensitivities (1, 2). Recent data from the CDC indicates a rising prevalence, reaching 1 in 31 8-year old children in 2025, with a notably higher frequency in males (3). In Uruguay, the last census from 2023 reports that 0.7% of the population is currently diagnosed with ASD (4).

The etiology of ASD is increasingly viewed through a multidimensional lens, acknowledging it as a result of dynamic interactions between polygenic risk and environmental features (42) including prenatal infections (5), gestational diabetes (6), and perinatal complications like hypoxia (7). In fact, many of these factors can act as critical modulators of genetic expression (1, 2). Beyond core behavioral symptoms, ASD is frequently associated with significant psychiatric and physiological comorbidities. Up to 70% of children with ASD present with concurrent conditions such as ADHD or anxiety (8), and between 23% and 70% experience gastrointestinal (GI) disorders (9). These GI issues are not only more prevalent in children with ASD than in neurotypical (NT) individuals but also correlate with increased irritability and behavioral severity (10, 11). This high prevalence has sparked intense interest in the gut-brain axis, where the intestinal microbiome is hypothesized to influence neurological processes through neuroactive metabolites (10).

Despite these associations, global ASD research suffers from a significant geographic bias; clinical models and public databases are overwhelmingly based on populations in developed nations, leaving Latin American cohorts heavily underrepresented (12). This gap is particularly detrimental in Uruguay, where systemic infrastructural barriers and the geographic centralization of specialists in Montevideo contribute to substantial diagnostic delays (13). Studies report an estimated 19–21 month gap between initial parental concerns and formal ASD diagnosis (14, 15). This delay is likely miltifactorial, caused both by the extreme geographic centralization, limited early identification tools and poor community-level awareness. Additionally, limited mental health resource allocation often restricts access to evidence-based treatments, forcing a reliance on interventions with less empirical support (16, 17).

Given these disparities, the primary objective of this study was to conduct a comprehensive clinical and sociodemographic characterization of a national cohort of 55 children with ASD and their neurotypical (NT) siblings. By implementing a sibling-matched design, we aimed to control for shared environmental and household factors to better understand the local manifestation of ASD in Uruguay. This work significantly expands upon our previous pilot study of 29 families (18), which focused on the interplay between gut microbiota and pharmacological treatments. In contrast, the current study enlarges the preliminary dataset to 55 families and shifts the focus toward a deep phenotypic characterization, while clarifying that microbiome analyses are part of a broader, future research phase and are not included in this manuscript. Our goals were: (1) to delineate the sociodemographic profile of these families and the systemic barriers they face; (2) to establish a comprehensive clinical and nutritional baseline for the Uruguayan ASD population; and (3) to generate the high-quality metadata necessary to support future longitudinal integration with gut microbiome data. These findings are essential for shortening the diagnostic gap and informing the development of personalized interventions tailored to the specific needs of children with ASD and their caregivers in Uruguay.

The early identification of Autism Spectrum Disorder (ASD) through standardized screening tools is a critical prerequisite for initiating timely support and improving long-term developmental trajectories (19). At a global scale validated instruments, such as the *Quantitative Checklist for Autism in Toddlers* (Q-CHAT) and the *First Year Inventory*, provide essential frameworks for detecting early deviations in communicative and social skills (20–22). In Uruguay these tools are infrequently used and remain unfamiliar to many professionals. Instead, local clinical practice primarily relies on the Child Development Surveillance Guide (23) developed by the Ministry of Public Health and UNICEF. This guide is intended for healthcare professionals and serves as a screening tool for developmental disorders. It provides information about children's developmental profiles at specific ages, as well as warning signs that may help identify developmental delays or deviations. It acts as a supportive resource for professionals assessing children's developmental achievements and communicating this information to their families. Despite this local framework, systemic infrastructural barriers contribute to substantial diagnostic delays. A delay in diagnosis is particularly detrimental because preemptive and early interventions have demonstrated significant effectiveness in optimizing social and behavioral outcomes when initiated during periods of high neuroplasticity (19, 24–29). To address these systemic disparities, the present study offers a novel multidimensional characterization of a national Uruguayan cohort. This characterization, framed within gastrointestinal symptomatology and nutritional habits, aims to improve the overall understanding of intestinal health in children with ASD, providing evidence that can be directly integrated into clinical practice. We envision that a better understanding of these factors will facilitate more accurate psychiatric referrals to appropriate professionals and strengthen overall intervention capacity by increasing clinical awareness.

## 2 Materials and methods

### 2.1 Participants selection criteria

A total of 110 children were recruited, 55 children with an ASD diagnosis with their cohabiting siblings without an autism diagnosis (NT). The project was approved by the Institutional Research Ethics Committee of the Pereira Rossell Hospital Center on August 11th 2022 (ID number 7281095). All families were orally informed of the project's aims and provided written informed consent prior to their participation. No financial compensation was provided to the participants; however, each family received an individualized written report detailing their child's dietary intake and nutritional results as a direct benefit of their participation. Both groups were between 4 and 10 years of age. A convenience sampling method was used for the selection of the cohort and thus this study should not be considered representative. Children with an ASD diagnostic were based on clinical assessment by a Child psychiatrist or a pediatric neurology specialist based on the Diagnostic and Statistical Manual of Mental Disorders, Fifth Edition (DSM-5) criteria (8). The Pediatric Psychiatry Academic Unit from Centro Hospitalario Pereira Rossell and Academic Unit of Pediatric Psychiatry, School of Medicine, University of the Republic (Montevideo, Uruguay) initiated contact with families. Recruitment was later expanded to include patients from other health providers and through social media. A database was then created with all children who met the proposed study criteria. Cohabiting siblings within the same age range were chosen as the control group to minimize differences in both genetic and environmental factors.

### 2.2 Recollection of clinical and sociodemographic data

In the clinical component of pediatric psychiatry, an exhaustive interview was conducted with a family member for each child, which was carried out in person or virtually, depending on each family's availability. Based on the interview, the researcher completed a structured form to collect comprehensive demographic and family data, including the child's sex, age, date of birth, geographic origin, health coverage, as well as the parents’ educational levels, work schedules, medical histories, and ages during pregnancy. The questionnaire also captured detailed pre-, peri-, and postnatal histories, documenting pregnancy complications, maternal pharmacological treatments, delivery method, gestational age, Apgar scores, perinatal complications, neonatal intensive care unit (NICU) admission, and neonatal anthropometry (birth weight, length, and head circumference). Early infancy and developmental milestones were evaluated by recording the type of lactation, sleep characteristics, infant temperament, the acquisition of walking before 18 months, first words before one year of age, sphincter control, and the presence of social smiling. Furthermore, general medical history was registered, noting any illnesses, hospitalizations, surgical interventions, epilepsy, electroencephalogram (EEG) alterations, and structural malformations. To assess core autism symptomatology, the interview documented any history of autistic regression and graded the severity (mild, moderate, or severe) of alterations in language, social interaction, restricted behaviors and interests, and autonomy. Additionally, atypical sensory responses were detailed across auditory, visual, olfactory, vestibular, tactile, and proprioceptive domains, alongside pain sensitivity. Finally, the diagnostic and educational trajectory was recorded, encompassing the age and origin of the first developmental concerns, the age of formal diagnosis, comorbid conditions, previous psychiatric or genetic evaluations, current schooling type and weekly schedule, educational adaptation, and the presence of a therapeutic companion.

### 2.3 Gastrointestinal symptoms

A standardized questionnaire was administered to participants to evaluate gastrointestinal symptoms following an interview as previously described (30) and adapted to spanish as in (18). Briefly, a questionnaire was completed by the children's caregivers or guardians to gather detailed information on the presence, frequency, and severity of various symptoms. The clinical variables assessed were: abdominal pain, abdominal distension, flatulence, diarrhea, constipation, defecation pain, food sensitivity, difficulty swallowing, presence of blood in the stool or vomit, and a previous diagnosis of food allergy. Additionally, we collected data on each participant's previous gastrointestinal diagnosis, feeding selectivity, and any current pharmacological treatments related to gastrointestinal symptoms.

### 2.4 Dietary intake

Dietary intake of foods and beverages over the 3 past months was assessed by research nutritionists from the School of Nutrition at the University of the Republic. Data were collected via interviews with the children's parents, using the SAYCARE food frequency questionnaire (FFQ) (31). This tool, validated for children and adolescents across seven cities in Latin America, was adapted in previous studies to incorporate foods typically part of a gluten-free and casein-free diet, which is relevant for individuals with autism (32). To estimate food portion sizes, a food photo booklet was shown to caregivers and children. The amounts of food consumed were recorded in household measures, which were then converted into grams or milliliters. Finally, based on the consumption frequency, the daily intake of each food was estimated. We compared the reported consumption frequencies of 17 distinct food items. The total sample size for this analysis was *n* = 45 families, as some participants opted out of completing the detailed food frequency questionnaire.

### 2.5 Statistical analysis

All data were processed and analyzed to characterize clinical, sociodemographic, and nutritional variables between the ASD and NT sibling groups. Categorical variables were expressed as frequencies and percentages, while continuous variables were expressed as mean ± standard deviation.

Differences in clinical and demographic categorical data between groups were assessed using the Fisher's Exact Test, which was chosen due to the presence of small expected frequencies in several symptom categories, providing a more robust alternative to asymptotic tests. To account for multiple testing across the nine evaluated gastrointestinal symptoms, the nominal *p*-values were adjusted using the Benjamini-Hochberg False Discovery Rate (FDR) method, with significance defined as an FDR-adjusted q-value < 0.05. Statistical computing, data processing, and visualizations were performed using the RStudio integrated development environment (version 2023.12.1) with R software (version 4.3.2).

For dietary intake given the non-normally distributed nature of the dietary frequency data, independent comparisons for each food category were conducted using the non-parametric Mann–Whitney U test. A *p*-value < 0.05 was set for statistical significance (two-tailed).

## 3 Results

### 3.1 Demographic data and family structure

**Figure 1:**
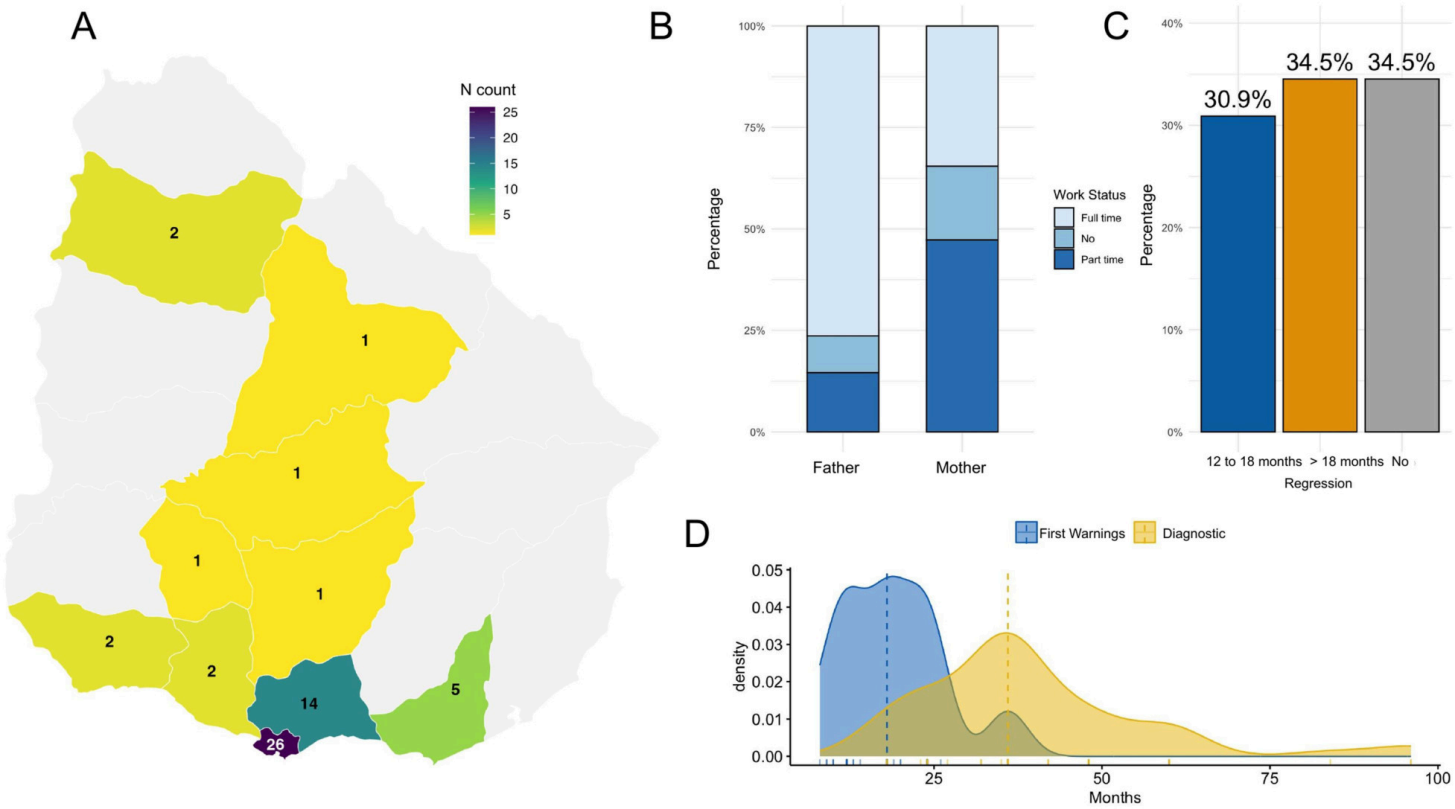
Family characteristics, region distribution and diagnostic characteristics in the Uruguayan cohort. **(A)** Number of families and its geographic distribution within Uruguay. **(B)** Paternal working hours for mothers and fathers. **(C)** Autistic regression reported by caregivers in months, **(D)** Difference between first reported warnings and diagnostic.

Our analysis of the study population identified a significant male-to-female ratio in children with Autism Spectrum Disorder (ASD), with 80% male and 20% female representation. In contrast, the neurotypical (NT) sibling group exhibited a more equitable sex distribution, comprising 47.3% males and 52.7% females (Table 1). Regarding geographic distribution, our cohort reflects the population density of Uruguay, with approximately half of the families (*n* = 26) residing in the capital, Montevideo, and half (*n* = 29) living in regions with lower population density (Figure 1A).

**Table 1:** Sex distribution in ASD and NT groups.

Group	Male %	Female %	*P* value
ASD children	80	20	< 0.001
Neurotypical siblings	47.3	52.7	< 0.001

*P*-values were calculated using Fisher's Exact Test.

We also identified a marked disparity in parental working hours. While 76.4% of fathers in our cohort were employed full-time, only 34.5% of mothers held full-time positions (Figure 1B). This pattern suggests that mothers in this cohort predominantly assume caregiving responsibilities, a finding consistent with broader regional data on caregiver strain in Latin America.

### 3.2 Prenatal factors and diagnostic trajectory

Pregnancy complications were reported in 60% of the pregnancies of children diagnosed with ASD, with 21.8% of these being high-risk multiple pregnancies (Table 2). The most frequent complications included threatened preterm labor, gestational diabetes, and maternal stress (evaluated through exposure to stressful life events). We observed no statistically significant differences in maternal or paternal age at birth between the ASD and NT groups (Table 3).

**Table 2:** Reported prenatal complications in the ASD group.

Prenatal Complication	n
Threatened preterm labor	22
Twin/Multiple pregnancy	12
Diabetes mellitus	6
Hypertension	5
Hematological complications	5
Maternal stress	5
Hypothyroidism	2
Cancer	2
Assisted reproductive technology	2
Placental abruption	1
Intrauterine growth restriction	1
Amniotic fluid abnormalities	1
Placenta previa	1

**Table 3:** Maternal and paternal age at the time of birth for each sibling.

Metric	ASD		NT		
	Maternal Age	Paternal Age	Maternal Age	Paternal Age	*P* value
Average	30,20	32,93	30,18	32,91	> 0.99
Standard deviation	4,83	6,89	5,52	7,12	> 0.99
Age range	21–39	18–54	19–40	19–55	-

*P*-values were calculated using Fisher's Exact Test.

Regarding developmental history, 65% of families reported that their children with ASD experienced autistic regression between 12 and 36 months of age (Figure 1C). Furthermore, there was a significant clinical lag, with a twenty-month gap between the appearance of the first warning signs and the formal ASD diagnosis (Figure 1D).

### 3.3 Birth comparative data for ASD and NT groups

Based on the three support levels categories defined in DSM-5, we classified the ASD group according to their support needs: we obtained 15% corresponded to mild ASD (Level 1), 58% to moderate ASD (Level 2), and 27% to severe ASD (Level 3). From the NT sample, we obtained birth data and anthropometric measurements for 38 children. The percentage of cesarean section deliveries was similar between groups, in the NT group was 68.4%, while in the ASD group it was 67.3%. Suggesting no association between delivery method and ASD diagnosis in this cohort. Likewise, no significant differences were observed in prematurity rates, with 28.9% of premature births in the group of children with ASD and 23.7% in the group of neurotypical siblings (Table 4). For the anthropometric data, we used the Modified Usher and Mc Lean tables for intrauterine growth in terms of weight, height, head circumference (HC) in cm, and percentiles in relation to gestational age. In relation to weight and height and head circumference, no differences were observed in both groups (Kendall Test not significant *p* > 0.05). Additionally, we documented early milestones for the ASD cohort, including independent walking and language acquisition, to further characterize this group (Table 5).

**Table 4:** Birth characteristics and anthropometric data.

Variable	Classification	ASD(%)	NT(%)	*P* value
Delivery	Vaginal delivery	0,342	0,316	0.843
	C-Section	0,658	0,684	0.843
Gestational age	Term	71,1%	76,3%	0.449
	Preterm	28,9%	23,7%	0.449
Weight	AEG	73,7%	81,6%	-
	GEG	21,0%	7,9%	-
	PEG	5,3%	10,5%	-
Heigth	p10-p90	68,4%	63,1%	0.638
	less than p10	23,7%	31,6%	0.638
	more than p90	7,9%	5,3%	0.638

*P*-values were calculated using Fisher's Exact Test.

**Table 5:** Early neurodevelopmental milestones in the ASD group.

Developmental Milestone	Percentage (%)
Independent Walking	
Achieved before 18 months	80.0%
Achieved after 18 months (Delayed)	20.0%
Language Acquisition (First Words)	
Achieved before 24 months	52.7%
Achieved after 24 months (Delayed)	47.3%

### 3.4 Gastrointestinal symptoms

We evaluated nine gastrointestinal (GI) symptoms to characterize the clinical profile of our cohort (Table 6). Children with ASD exhibited a significantly higher burden of specific lower gastrointestinal issues compared to their NT siblings. After adjusting for multiple comparisons using the Benjamini-Hochberg FDR method, four symptoms remained significantly more prevalent in the ASD group: meteorism (q = 0.002), abdominal distension (q < 0.001), diarrhea (q = 0.001), and abdominal pain (q = 0.011). Other symptoms, such as constipation, vomiting, and blood in the stool, did not show statistically significant differences. These findings underscore a distinct GI symptom profile in this cohort that persists even when controlling for the shared household environment.

**Table 6:** Gastrointestinal symptoms and previous GI diagnosis in both groups.

Gastrointestinal Symptoms	ASD (*n* = 55)	NT (*n* = 55)	Nominal *p*-value	Adjusted q-value (FDR)
Abdominal distension*	27	5	*p* < 0.001	q < 0.001
Diarrhea*	24	6	*p* < 0.001	q = 0.001
Meteorism*	36	17	*p* < 0.001	q = 0.002
Abdominal pain*	22	8	*p* = 0.005	q = 0.011
Constipation	22	16	*p* = 0.316	q = 0.569
Defecatory pain	12	11	*p* > 0.999	q > 0.999
Vomiting	6	4	*p* = 0.742	q = 0.954
Blood in stool	6	4	*p* = 0.742	q = 0.954
Blood in vomit	0	0	*p* > 0.999	q > 0.999

*P*-values were calculated using Fisher's Exact Test. The adjusted q-values were calculated using the Benjamini-Hochberg FDR method to correct for multiple testing across the 9 symptoms.

*Statistically significant, *p* < 0.05.

### 3.5 Dietary consumption patterns

We compared the consumption frequencies of 17 distinct food items between the ASD and NT groups (Table 7). While initial unadjusted analyses suggested lower frequencies of milk (*p* = 0.017) and yogurt (*p* = 0.014) consumption in the ASD group, these differences did not remain statistically significant after applying the Benjamini-Hochberg FDR correction (all q > 0.05). These results suggest that the overall consumption frequencies of major food groups do not diverge significantly within our sibling-matched cohort, likely reflecting the shared dietary environment of the household.

**Table 7:** Food intake according to neurodevelopment.

Food Intake	ASD (*n* = 45)	NT (*n* = 45)	U de Mann–Whitney	*p*-value
Beans	2.73 ± 6.03	2.20 ± 5.13	1008,5	0.968
Lentils	8.47 ± 13.72	6.39 ± 8.20	958,5	0.654
Chickpeas	3.96 ± 10.46	3.08 ± 6.29	996	0.875
Total Legumes	15.17 ± 24.17	11.69 ± 14.85	958,5	0.659
French fries	18.40 ± 23.85	11.84 ± 13.18	900,5	0.358
Cooked potato	18.40 ± 23.85	11.63 ± 11.78	900,5	0.357
Sweet potato	13.61 ± 18.92	11.93 ± 17.80	985	0.814
Raw vegetables	19.32 ± 33.97	20.64 ± 31.20	871,5	0.240
Cooked vegetables	332.81 ± 881.71	215.08 ± 449.68	925	0.458
Fruit	550.37 ± 548.78	387.72 ± 386.32	853	0.192
Milk*	169.66 ± 270.10	252.45 ± 246.56	728	0.017*
Yogurt*	31.12 ± 105.12	47.93 ± 107.15	753	0.014*
Meat	26.00 ± 31.12	29.13 ± 19.88	800,5	0.076
Chicken	32.23 ± 30.19	28.75 ± 17.70	861,5	0.836
Pork	15.35 ± 13.34	13.34 ± 20.85	968	0.707
Fish	8.72 ± 12.25	9.29 ± 11.93	931	0.497
Sweets/Candies	2.38 ± 4.48	3.02 ± 4.41	832	0.133

*P*-values were calculated using Mann–Whitney U test.

*Statistically significant, *p* < 0.05.

## 4 Discussion

The primary objective of this study was to provide a comprehensive clinical, sociodemographic, and nutritional characterization of a national cohort of 55 Uruguayan children with Autism Spectrum Disorder (ASD) and their neurotypical siblings. This research specifically addresses the geographic underrepresentation of Latin American populations in ASD literature while offering local data to help understand and eventually reduce the significant diagnostic gap observed in Uruguay.

Our findings reflect a male-to-female ratio of 4:1, which is consistent with global epidemiological trends. Notably, the study reveals significant socioeconomic impacts on family structures; fathers showed higher full-time employment rates (76.4%) compared to mothers (34.5%), who predominantly assumed caregiving roles and participated in 98% of study interviews, underscoring the disproportionate physical and emotional burden on mothers (33). These results align with REAL network data indicating that Latin American caregivers face substantial financial strain and employment loss (12).

Environmental modulators may play a role in the clinical landscape of this cohort (1). We observed a high rate of multiple pregnancies (21.8%) and pregnancy complications (60%), both significantly exceeding Uruguayan national averages. Perinatal complications, including preterm birth (21.8%) and fetal hypoxia (9.1%), further support the link between early biological stress and ASD (7). Previous multicenter research has demonstrated that while the distribution of head circumference in individuals with Autism Spectrum Disorder (ASD) is generally normal, it exhibits an increased mean and variance, alongside an elevated rate of macrocephaly (without a corresponding increase in microcephaly) (34). Furthermore, this literature indicates that head circumference in autistic individuals tends to be larger relative to height, and that larger head size correlates with greater severity in social skill deficits and delayed language onset (34). Based on this background, we evaluated these clinical variables within our sibling-matched cohort of 55 families. When comparing the two groups, we observed a trend toward larger head circumferences at the moment of birth in children with ASD compared to their neurotypical siblings; however, this difference did not reach statistical significance. Within the ASD cohort itself, we observed a high percentage of head circumferences at birth above the 90th percentile, which aligns with existing literature (35). Nevertheless, we did not find statistically significant associations between head circumference and language acquisition delays, social skills, restricted interests and behaviors, or overall disorder severity. This lack of significance, coupled with the high variability observed in our data, likely reflects the limited statistical power of our sample size while further underscoring the complex clinical heterogeneity characteristic of ASD.

A critical finding is the 20-month diagnostic gap between the first parental concerns (mean 19 months) and formal diagnosis (mean 39 months). This delay persists despite the fact that 60% of signs emerge before age two (36). Furthermore, our sample showed a remarkably high rate of autistic regression (65%), significantly higher than the 15.6%–27% reported in epidemiological literature (37–39), likely reflecting the specific clinical nature of our cohort (43).

In terms of physical health, 94.3% of children with ASD presented with gastrointestinal (GI) symptoms, a significant increase compared to their neurotypical siblings (60.3%; *p* < 0.001). This high GI burden corroborates and strengthens the overall clinical trend first identified in our initial cohort of 29 families (18). Over 78% of the ASD group suffered from transit disorders like diarrhea or constipation. Interestingly, while food selectivity was prevalent (64.6%), it did not significantly correlate with the type or number of GI symptoms. Regarding dietary intake, our results suggested a lower consumption of milk and yogurt in the ASD group. This is clinically relevant as it may reflect the common adherence to gluten-free or casein-free (GFCF) diets in the ASD community. This reduced dairy intake may be hypothesized to reflect the widespread, often unsupervised, adoption of casein-free diets within the ASD community. Current clinical practices in Latin America often see families implementing restrictive diets based on anecdotal evidence of behavioral improvement, despite a lack of global scientific consensus supporting such interventions for all children on the spectrum. These findings are consistent with those previously reported by Mendive et al. and could be linked to the adherence to casein-free diets that have become popular in this population, despite the lack of scientific consensus to support such interventions (32, 40). Furthermore, while food selectivity was prevalent in 64.6% of our sample, it did not significantly correlate with the type or number of GI symptoms. Sensory processing alterations were universal in our ASD group (100%), echoing findings by Ben-Sasson et al. (41) that highlight sensory symptoms as a core feature of the disorder (41).

The lack of strong correlation between specific food items and clinical severity markers underscores the complexity of the gut-brain axis. It is possible that the impact of nutrition on ASD symptoms is mediated by individual microbial metabolism rather than the gross intake of specific food categories.

These findings strongly advocate for an interdisciplinary approach to pediatric care that integrates early GI screening alongside standard neurodevelopmental evaluations. Furthermore, the 20-month diagnostic delay observed in this population highlights the need to improve diagnostic tools and interventions. Ultimately, this comprehensive clinical and nutritional baseline provides the necessary background for our upcoming integration of full-cohort microbiome data, which will further help to understand the complex gut-brain axis and drive the development of context-specific, personalized therapeutic strategies for Latin American populations.

## 5 Strengths and limitations

The authors acknowledge that this study has certain limitations. Primarily, the use of a convenience sampling method and the size of the cohort (55 families) limits the generalizability of the findings on a national level and reduces the statistical power for complex subgroup analyses. Nonetheless, this study represents a pioneering interdisciplinary effort in Uruguay, bridging gaps between clinical practice and biological research to better understand neurodevelopmental conditions.

Regarding clinical implications, the results obtained suggest that the primary utility of this characterization lies in facilitating timely referrals. In Uruguay, the use of the Child Development Surveillance Guide (23) is an established practice for initial screening. However, our work provides evidence for the importance of a surveillance approach that includes common associated comorbidities. The identification of a high prevalence of gastrointestinal symptoms (94.3%) and alterations in dietary behavior (64.6%) in children with ASD underscores the need for the early referral of these patients to gastroenterology and nutrition specialists. This integrated approach aims to improve comprehensive intervention capacity and the quality of life for these children by addressing comorbidities that significantly impact their daily well-being.

Finally, this study establishes a critical clinical and nutritional baseline for the future integration of gut microbiome metagenomic data. In the future, this information may contribute to more holistic diagnostic assessments of autism and the development of personalized, contextualized tools for the understudied Latin American population.

## 6 Conclusions

This study provides an essential clinical and sociodemographic characterization of Autism Spectrum Disorder (ASD) in Uruguay, contributing vital regional data to a field often underrepresented in global literature. Our findings reveal critical systemic and physiological challenges, most notably a significant 20-month diagnostic gap between initial parental concerns and formal diagnosis. This delay, coupled with a high rate of autistic regression (65%), underscores the urgent need to strengthen local early detection strategies and improve access to specialized neurodevelopmental assessments.

Physiologically, the high prevalence of gastrointestinal symptoms (94.3%) identified in the ASD group highlights the necessity of an interdisciplinary pediatric approach that integrates GI screening into standard care. Furthermore, the observed socioeconomic disparities in family structures, where caregiving responsibilities and study participation fell more on mothers (98%), point to a significant family burden that requires targeted social support systems.

While our current results on specific risk factors remain exploratory given the cohort size, the deep phenotypic and nutritional baseline established here is a pivotal first step. This comprehensive metadata will serve as the foundation for future integration with gut microbiome data, moving toward a more holistic understanding of the gut-brain axis and the development of personalized therapeutic tools tailored to the specific needs of children with ASD and their caregivers in Uruguay.

## Data Availability

The raw data supporting the conclusions of this article will be made available by the authors, without undue reservation.
